# Hidden blood loss and risk factors after percutaneous endoscopic transforaminal lumbar interbody fusion

**DOI:** 10.3389/fsurg.2025.1490038

**Published:** 2025-01-15

**Authors:** Meng Ge, Fangbing Zhu, Weibin Du, Zhengcong Ye, Zhenfei Xiong, Lukai Zhang, Hua Zhou, Jun Yang

**Affiliations:** ^1^ Research Institute of Orthopedics, The Affiliated Jiangnan Hospital of Zhejiang Chinese Medical University, Hangzhou, China; ^2^ Hangzhou Xiaoshan Hospital of Traditional Chinese Medicine, Hangzhou, China

**Keywords:** percutaneous endoscopic transforaminal lumbar interbody fusion, hidden blood loss, minimally invasive spine surgery, lumbar degenerative diseases, risk factors

## Abstract

**Purpose:**

In this study, we aimed to assess the occurrence of hidden blood loss (HBL) and its associated risk factors in patients with lumbar degenerative diseases who underwent percutaneous endoscopic transforaminal lumbar interbody fusion (Endo-TLIF).

**Methods:**

Sex, age, height, weight, body mass index, and medical history including hypertension, diabetes, and osteoporosis were recorded. The duration of symptoms, preoperative lumbar subcutaneous fat tissue thickness (measured using midsagittal T2-weighted magnetic resonance imaging), lumbar disc degeneration grade, and other basic patient information were also documented. The levels of fibrinogen, activated partial thromboplastin time, prothrombin time, thrombin time, and platelet count as well as the pre- and postoperative hematocrit and hemoglobin levels were collected. In addition, the number of fusion levels, surgical time, and intraoperative blood loss were recorded. Total blood loss (TBL) was calculated using the gross formula, and HBL was calculated based on the TBL and visible blood loss. The risk factors were analyzed using single-factor correlation and multivariate linear regression analyses.

**Results:**

Of the 83 patients, there were 42 males and 41 females. Hypertension (*P* = 0.003), fusion level (*P* < *0.001*), and surgery time (*P* < *0.001*) were significantly correlated with HBL via a single-factor correlation analysis. Multiple linear regression analysis showed that the fusion level (*P* < *0.001*) and surgery time (*P* < *0.001*) were independent risk factors for HBL.

**Conclusion:**

In patients with lumbar degenerative diseases treated with Endo-TLIF, HBL accounts for a large proportion of TBL. A large number of fusion segments and prolonged operation time are risk factors for increased perioperative HBL during Endo-TLIF. Increased attention should be paid to the presence of HBL to ensure the safety of perioperative patients.

## 1 Introduction

Despite significant progress in spinal fusion surgery over the past several years, the pursuit of minimally invasive spine surgery is ongoing. Lumbar spine fusion remains an efficient surgical method for treating various lumbar degenerative diseases, including spondylolisthesis and spinal stenosis (1–3). The percutaneous endoscopic transforaminal lumbar interbody fusion technique, also known as Endo-TLIF, has emerged significantly in recent years, drawing the attention of spine surgeons owing to its benefits, such as reduced trauma, less bleeding, conservation of soft tissues, quicker recovery rate, short duration of hospital stay, and better clinical results (4–6). In 2000, Sehat et al. were the first to describe the notion of hidden blood loss (HBL). They discovered that it comprised 26% and 49% of total blood loss (TBL) following total knee and hip replacement operations, respectively (7). Subsequent studies have shown that HBL contributes to increased TBL and risk of postoperative blood transfusion if not properly managed (8, 9). Furthermore, elevated HBL levels can result in extended periods of hospital stay and recuperation, slow progress in wound recovery, heightened susceptibility to infections, and increased risk of cardiovascular and cerebrovascular accidents due to augmented bleeding volume (10, 11). Given the growing concern among spinal surgeons regarding HBL, an increasing number of researchers have investigated this issue (12–14). However, despite the expanding literature on HBL and minimally invasive spine surgery, no study has yet been conducted to quantify HBL and analyze its risk factors specifically in the context of Endo-TLIF. *Ignorance of hidden blood loss after Endo-TLIF can lead to several complications in patients due to increased blood loss, such as anemia, increased rates of postoperative infections, prolonged hospital stays, and slow incision recovery.* To address this, we carried out a retrospective study to determine the amount of HBL in Endo-TLIF procedures and investigate the associated risk factors. Our aim was to provide theoretical guidance to spinal surgeons performing Endo-TLIF to prevent related complications.

## 2 Materials and methods

### 2.1 Patients

Between Oct 2017 and Mar 2021, 83 individuals with lumbar degenerative disease underwent surgery using the Endo-TLIF technique at the Spine Surgery Department of the hospital, where the primary author worked. This study included 42 males and 41 females. *The inclusion criteria were as follows: patients at least 18 years old; confirmed lumbar spinal stenosis; mild lumbar spondylolisthesis (Meyerding grades I and II) with symptoms of recurrent lumbar disc herniation; symptoms that did not improve or worsened after receiving 6 mo or more of conservative treatment; and patients with segmental instability. The exclusion criteria included patients unable to undergo surgery due to severe systemic disease; those with severe lumbar spondylolisthesis (Meyerding grade Ⅲ and Ⅳ); patients with hematologic diseases; patients using antiplatelet drugs or anticoagulants; and those with lumbar spine tumor, tuberculosis, or infection.* In this research, a single senior surgeon, who had already reached the pinnacle of his learning curve, performed all the surgeries. The ethics committee of the hospital approved this study and ensured that all procedures adhered to the necessary regulations and guidelines.

### 2.2 Data collections

The authors’ Hospital provided all patient information through a digital health database. This included demographic characteristics, such as age, sex, body mass index (BMI), height, and weight. Details of the duration of symptoms, smoking and drinking habits, hypertension, diabetes, osteoporosis, thickness of preoperative subcutaneous fat tissue in the lumbar region (as measured using midsagittal T2-weighted MRI) (15), and grade of lumbar disc degeneration (according to Pfirrmann grades) were also included (16). The parameters associated with blood loss, such as the patient blood volume (PBV), TBL, preoperative fibrinogen (FIB), activated partial thromboplastin time (APTT), prothrombin time (PT), thrombin time (TT), platelet count (Plts), intraoperative blood loss, and pre- and postoperative hematocrit (Hct) and hemoglobin (Hb) levels, were important considerations. Furthermore, operative data such as the number of fusion levels, duration of the surgery, and intraoperative blood loss were recoded. The World Health Organization defines anemia as hemoglobin (Hb) levels below 12.0 g/dl in women and below 13.0 g/dl in men (17, 18). All data were collected from the electronic medical record system.

### 2.3 Surgical procedures for endo-TLIF

All patients underwent general anesthesia and were placed in the prone position, with their abdomens lifted to minimize lumbar lordosis. The lesion segment was identified using C-arm fluoroscopy, and the locations of the surgical channel of the intervertebral foramen and skin cut of the pedicle screw were marked. A decompression cut approximately 1 cm in length was created approximately 4.5–5 cm from the midline of the surgery area. This incision is utilized for decompression, fusion, and pedicle screw placement. Its length can be adjusted to allow for parallel interbody decompression and cage placement, and from the perspective of the C-arm, the working cannula is positioned within the intervertebral foramen, while the display and light source are linked together. The joint capsule was treated with endoscopic electrocoagulation and the facet joint and part of the vertebral plate were gradually removed under endoscopic guidance using a circular saw.

Upon removal of the ligamentum flavum, the nerve roots and dural sac of the responsible segment were exposed. The large cannula was replaced, and the intervertebral space was treated with a reamer to scrape the cartilage endplate of the diseased segment. This method prepares the surface for the implant fusion under endoscopic guidance. A test mold is used to select the fusion cage of appropriate size. A homemade iliac bone extractor is used to collect cancellous bone from the posterior superior iliac spine. This procedure was performed after a cut was made in the contralateral distal arch of the nail tract. The bone is then implanted into the vertebral space of the responsible segment along with decompressed bone from the articular eminence and vertebral plate. When enough bone graft has been obtained, a fusion cage filled with autogenous bone is inserted parallel to the vertebral space using an expanded transforaminal method. C-arm fluoroscopy was performed to ensure that the cage remained in satisfactory position. The nerve roots on the affected side of the responsible segment were reexamined under endoscopic guidance. Finally, pedicle screws and bilateral connecting rods were placed percutaneously, and the skin was sutured and covered with a sterile dressing after confirming the proper position for internal fixation using C-arm fluoroscopy.

### 2.4 Postoperative managements

Postoperatively, all patients were on bed rest and received a rehydration regimen comprising of hormones, pain medications, and electrolytes. No anticoagulants were administered; however, the patients were instructed to move and raise their legs in bed and receive pneumatic pump therapy to prevent thrombosis. The patients were allowed to walk in the ground after confirming that the implant was in a satisfactory position via an imaging review. No drains were inserted; therefore, monitoring of drainage was not required.

### 2.5 Calculation of hidden blood loss

HBL is equal to TBL minus visual blood loss (VBL) plus transfusion, that is, HBL (ml) = TBL−VBL + transfusion. Therefore, to calculate HBL, we need to calculate TBL and VBL. For calculating TBL, formula by Gross et al.is used, which is given by TBL (ml) = [PBV (L) × (Hct_pre_−Hct_post_)]/[(Hct_pre_ + Hct_post_)/2] × 1,000 (19). According to the formula guiding the patient's Hct, we also choose the preoperative and Hct after blood volume stabilization for 23 d postoperatively. Nadler et al. considered the patient's complete blood volume, suggesting that it can be calculated based on the patient's sex, height, and weight. The formula PBV (L) is equal to k1 multiplied by the cube of height (m) plus k2 multiplied by weight (kg) plus k3. For males, the values of k1, k2, and k3 were 0.3669, 0.03219, and 0.6041, respectively. For females, these constants were 0.3561, 0.03308, and 0.1833, respectively. It is important to mention that all patients did not have drains postoperatively, hence, the VBL is approximately equal to the intraoperative blood loss. The calculation of intraoperative blood loss considers the weight of blood found in the suction bottle (while eliminating the weight of the irrigation fluid used during surgery), as well as the blood in the gauze and gauze strips (eliminating the weight of the dry gauze and gauze strips used during surgery). Additionally, none of the patients required intraoperative or postoperative transfusion, leading to the equation as follows: HBL = TBL—intraoperative blood loss. From the above statement, to obtain the amount of HBL, we only needed to calculate the TBL based on the change in Hct and calculate the VBL based on the intraoperative blood loss. The difference between the TBL and intraoperative blood loss equal to the HBL.

### 2.6 Statistical analysis

We utilized SPSS v26.0 for Windows (IBM Corp., Armonk, NY, USA) for data analysis. All quantities were derived as mean ± SD. The level of statistical significance was set at *P* < 0.05. All variables that might be related to HBL were screened using single-factor correlation analysis. It included 14 quantitative variables (age, BMI, duration of symptoms, lumbar subcutaneous fat tissue thickness, time of surgery, preoperative FIB, APTT, PT, TT, Plts, preoperative Hct and HB, and postoperative Hct and HB two days after surgery) and 9 qualitative variables (sex, hypertension, diabetes, osteoporosis, smoking, drinking, anemia, disk degeneration grade, and number of fusion levels). Sex, hypertension, diabetes, osteoporosis, smoking, and drinking were analyzed using the independent sample *t* test, and disk degeneration grade and number of fusion levels were analyzed using one-way analysis of variance test. Age, BMI, duration of symptoms, time of surgery, lumbar subcutaneous fat tissue thickness, preoperative FIB, APTT, PT, TT, and Plts were analyzed by Pearson or Spearman correlation analysis. Variables with a significant correlation were selected, and multivariate linear regression analysis was used to identify independent risk factors associated with HBL. A positive correlation coefficient implies a direct relationship with HBL, whereas a negative coefficient indicates the same relationship. Discrepancies in preoperative and postoperative Hct and Hb levels were examined using Student's *t*-test. Differences between pre- and postoperative anemia were assessed using the chi-square test.

## 3 Results

In our study, 83 patients (42 males and 41 females) underwent Endo-TLIF surgery. *The average age was 58.6* *±* *9.9 years* (*35–79*) *and average BMI was 24.5* *±* *2.8 (18.5–31.3)*. The demographic information is presented in Table 1. The Hct and Hb preoperative readings were 0.412 ± 0.04 and 140 ± 15 g/L respectively, whereas the postoperative readings were 0.338(*P* *<* *0.001*) and 110 ± 16(*P* *<* *0.001*), respectively. Meanwhile, 58 patients developed anemia after surgery (*P* *<* *0.001*, Table 2). The findings of the single-factor correlation analyses are presented in Table 3. Hypertension (i.e., blood pressure ≥140/90 mmHg) (*P* = 0.003), fusion levels (*P* = 0.003), and surgery time (*P* *<* *0.001*) were significantly correlated with HBL through single-factor correlation analysis. However, the factors such as sex (*P* = 0.597), age (*P* = 0.420), BMI (*P* = 0.860), diabetes (i.e., fasting blood-glucose ≥6.1 mmol/L) (*P* = 0.809), osteoporosis (i.e., Dual-emission x-ray absorptiometry, T value ≤–2.5) (*P* = 0.219), smoking (*P* = 0.103), drinking (*P* = 0.978), Pfirrmann grades (*P* = 0.150), duration of symptoms (*P* = 0.795), preoperative lumbar subcutaneous fat tissue thickness (*P* = 0.867), preoperative FIB (*P* = 0.666), APTT (*P* = 0.778), TT (*P* = 0.650), PT (*P* = 0.820), and Plts (*P* = 0.373) were not correlated with HBL. Multiple linear regression analysis revealed a significant correlation between fusion level (*P* *<* *0.001*) and surgery time (*P* *<* *0.001*) with HBL (Table 4).

**Table 1:** Patients demographics.

Parameters	Statistics
Total patients (*n*)	83
Sex (*n*)	
Female	41
Male	42
Age, year	58.6 ± 9.9
BMI, kg/m^2^	24.5 ± 2.8
Duration of symptoms(day)	113.5 ± 111.8
Hypertension (*n*)	
No	39
Yes	44
Diabetes (*n*)	
No	69
Yes	14
Osteoporosis	
No	73
Yes	10
Smoking (*n*)	
No	59
Yes	24
Drinking (*n*)	
No	64
Yes	19
Pre-operative Lumbar subcutaneous fat tissue thickness (mm)	13.8 ± 7.8
Pfirrmann grades	
C	4
D	62
E	17
Fusion level (*n*)	
1	66
2	14
3	3
Surgery time, (min)	241 ± 34.5
PT, s	11.1 ± 0.6
APTT, s	26.0 ± 2.5
TT, s	17.6 ± 1.3
FIB,g/L	2.7 ± 0.6
PLT, g/L	208.4 ± 51.2
Preoperative Hct	0.412 ± 0.04
Preoperative Hb, g/L	140 ± 15
Postoperative Hct	0.338 ± 0.04
Postoperative Hb, g/L	110 ± 16
TBL, ml	880.7 ± 219.8
VBL, ml	60.8 ± 17.6
HBL, ml	819.8 ± 204.9

**Table 2:** Changes in Hct, Hb, and Anemia level following endo-TLIF spine surgery.

	Preoperative (*n* = 83)	Postoperative (*n* = 83)	F	*P*
Hct	0.412 ± 0.04	0.338 ± 0.04	0.012	0.000
Hb, g/L	140 ± 15	110 ± 16	0.184	0.000
Anemia	9	67	81.641	0.000

**Table 3:** Results of single factor correlation analysis for HBL.

Index	Groups	*n*	HBL (ml)	t/F	*P*
Sex					0.597
	Female	41	832.0 ± 218.6	0.680	
	Male	42	808.0 ± 192.6		
Hypertension				4.611	0.003
	with	41	886 ± 225.4		
	without	42	775.1 ± 160.6		
Diabetes				0.181	0.809
	with	14	831.9 ± 228.1		
	without	69	817.4 ± 201.7		
Osteoporosis				0.061	0.219
	with	10	744.7 ± 243.7		
	without	73	830.1 ± 198.8		
Smoking				1.929	0.103
	with	24	877.4 ± 234.2		
	without	59	796.4 ± 188.9		
Drinking				2.278	0.978
	with	19	818.7 ± 163.2		
	without	64	820.2 ± 216.9		
Pfirrmann grades				1.940	0.150
	C	4	1,009.8 ± 240.8		
	D	62	805.1 ± 196.7		
	E	17	829.0 ± 216.5		
Fusion segaments				118.9	0.000
	1	66	773.4 ± 111.4		
	2	14	1,108.8 ± 64.8		
	3	3	1,372.8 ± 204.9		
	r				*P*
Age	0.09				0.420
BMI	0.02				0.860
Duration of symptoms(day)	0.029				0.795
Pre-operative Lumbar subcutaneous fat tissue thickness	−0.019				0.867
Surgery time, min	0.87				0.000
PT, s	−0.025				0.820
APTT, s	0.031				0.778
TT, s	0.05				0.650
FIB,g/L	0.066				0.666
PLT, g/L	−0.099				0.373

**Table 4:** Results of multiple line regression analysis for HBL.

Index	Unstandardized		Standardized	T	*P*	VIF
	B	SE				
Hypertension	11.001	19.625	0.027	0.561	0.577	1.121
Fusion levels	185.866	30.203	0.461	6.154	0.000	2.271
Surgery time, min	2.944	0.405	0.495	6.542	0.000	2.767

## 4 Discussion

Degenerative lumbar spine disease is common in older patients and frequently causes symptoms such as lower back pain, shooting pain in the lower limbs, and periodic limping. These can lead to decreased movement, possible disability, and substantial reduction in the patient's quality of life. An efficient method to treat such degenerative conditions of the lumbar spine is through a procedure known as lumbar interbody fusion. It directly decompresses the nerve roots, corrects lumbar lordosis, and indirectly restores the height of the lumbar spinal space, ultimately achieving cure.

While traditional open procedures, such as PLIF and TLIF, yield positive results, they are often poorly tolerated by many older patients with underlying health conditions. Open surgery requires large incisions, muscle stripping, and pulling, increasing the risk of complications such as excessive blood loss, extended postoperative recovery time, and higher chances of postoperative infections. Recently, more spine surgeons have turned to Endo-TLIF surgery, a less invasive operation that causes less bleeding and involves small incisions with a cannula-protected operation, offering improved protection for the soft tissues, reducing postoperative infection risk, and promoting quick recovery. Existing literature suggests that Endo-TLIF effectively alleviates pain in older patients with poor underlying conditions who struggle with open surgery, significantly improving their quality of life (14, 20–22).

HBL is a condition where bleeding is undetectable during surgery. Traditionally, physicians have relied on estimates and formula calculations to determine blood loss. However, research conducted by Sehat et al. indicated that this technique is susceptible to a considerable number of errors. He demonstrated how postoperative blood can seep into muscle gaps and underlying compartments, leading to hidden bleeding that cannot be measured accurately, referred to as HBL (7).

HBL poses serious risks to patient recovery and safety after surgery. Underestimating blood loss can result in postoperative anemia, which in turn can weaken the patient's strength and immunity and increase the risk of infection and complications. Moreover, the healing process of wounds may be hindered by covert blood loss, which contributes to leakage of blood into tissue areas, consequently increasing the possibility of infection and development of scars. Furthermore, it can lead to prolonged postoperative hospitalization and increased utilization of healthcare resources.

Consequently, surgeons have begun to focus on addressing concealed blood loss. An increasing number of scholars have discovered that even in minimally invasive surgery, HBL cannot be ignored (Zhou et al. noted a substantial HBL (488.4 ± 294.0 ml, 52.5% of TBL) post MIS-TLIF treatment (12). Similarly, an average hidden blood loss of (317 ± 156) ml was discovered in 125 osteoporotic vertebral compression fracture patients treated with percutaneous vertebroplasty (23). Wang et al. reported notable HBL of approximately 469.5 ± 195.3 ml following UBE surgery, constituting 57.6% of TBL (13). Additionally, patients who underwent anterior cervical discectomy fusion experienced a substantial HBL of approximately 487.98 ± 255.96 ml, making up 67.61 ± 5.20% of the TBL (24). However, there is currently a lack of research on HBL in Endo-TLIF, preventing the calculation of HBL and analysis of risk factors. This knowledge gap hinders the effective management of patients undergoing Endo-TLIF. To resolve this problem, we conducted a retrospective study on HBL and its risk factors in patients who underwent Endo-TLIF. Through this, we intended to offer theoretical assistance for postoperative care and minimize the complications caused by excessive blood loss. This study included various risk factors such as sex, age, BMI, hypertension, diabetes, osteoporosis, smoking, drinking, Pfirrmann grades, fusion segments, symptom duration, preoperative lumbar subcutaneous fat tissue thickness, operation time, preoperative FIB, PT, APTT, TT, and Plts. In this study, we found that the HBL in patients treated with Endo-TLIF was 819.8 ± 204.9 ml. Single-factor correlation analysis revealed that increased blood pressure, operation time, and fusion segment were associated with high HBL. Multiple linear regression analysis confirmed that the duration of operations and fusion segments independently contributed to the risk of HBL, consistent with the findings of Zhang (25). Long operation times may result in increased HBL, possibly owing to prolonged washing time, extended wear time of the wound surface, and more severe inflammatory reactions, leading to increased hemolysis. This finding is consistent with the conclusions of Wang et al. and Lei et al (13, 26). Prolonged surgery may involve greater tissue cutting and incision widening, leading to increased blood vessel damage and HBL. It can also cause localized tissue edema, further increasing the risk of HBL (26). Analysis of the increase in HBL associated with the fusion segments revealed that an increase in the fusion segments led to greater soft tissue damage and bone destruction. With more fusion segments, additional tissue spaces and surgical cavities were created, increasing the blood storage capacity of HBL. Furthermore, bone debris from multi-level decompression and intervertebral space treatment may enter the bloodstream, causing abnormal capillary bed opening and blood extravasation, thereby increasing HBL. Removing the joints and ligaments located between the disc and vertebral body affects the blood flow to the fused area, reducing blood supply and leading to ischemia and concealed hemorrhage within the fused section. This finding aligns with the conclusions of Yu et al. (27). In our study, we discovered that factors such as sex, age, BMI, hypertension, diabetes, osteoporosis, smoking or drinking habits, Pfirrmann grades, symptom duration, prior lumbar subcutaneous fat tissue density, preoperative FIB, PT, APTT, TT, and Plts examinations had no substantial correlation with HBL. These results contradict those of the previous studies. Wang et al. identified age and preoperative FIB as risk factors in patients undergoing UBE surgery (13), while Zhou et al. revealed that age, muscle thickness, and preoperative FIB were independent risk factors for HBL in MIS-TLIF (12). Dai found that diabetes and hypertension are risk factors for HBL, which may be due to varying surgical procedures. Our Endo-TLIF procedure involves four small incisions of approximately 1 cm for decompression, fusion, and screw placement. This approach minimizes soft tissue damage and handles cartilage endplates under direct endoscopic visualization to reduce bone damage, thus decreasing HBL and mitigating risk factors associated with it. Moreover, the variety of patient populations with differing types and severity of conditions that various studies may incorporate can cause disparities in outcomes. In our study, the TBL of Endo-TLIF was 880.7 ± 219.8 ml. Some scholars have suggested that the use of new bone materials, such as biologics or allografts, may reduce TBL (28). Nevertheless, some studies have suggested that autologous iliac bone is the preferred graft for spinal fusion surgery. Consequently, we continued to extract the iliac bone during the surgery. However, this procedure can lead to concealed blood loss, and an overall increase in blood loss (29–31). Therefore, it is crucial to use bone wax and other techniques to effectively control bleeding after bone removal.

Our study has several limitations. Primarily, this was a retrospective study that focused on a single center and involved a small number of patients. The included risk factors are not comprehensive enough. To mitigate the risk of false positives and bias, we need to execute a prospective, multicenter study with a large sample size in the future to enhance the accuracy of the results. These factors likely influenced our findings. Secondly, we used Hct levels measured 2–3 d postoperatively, but it was still not possible to determine the balance of blood circulation, potentially causing errors in the amount of HBL calculated using the formula. Lastly, all our surgical cases were performed during the platform stage of the surgeons’ operations, ignoring the surgery-related situation of spine surgeons at the rising stage of the learning curve; therefore, the correlation between surgical experience and HBL was not addressed.

## 5 Conclusion

To summarize, a significant percentage of the TBL in patients with lumbar degenerative diseases treated with Endo-TLIF can be attributed to HBL. An increased number of fusion segments and prolonged operation time are risk factors for increased perioperative HBL during Endo-TLIF. Attention to HBL is critical for ensuring patient safety during perioperative care.

## Data Availability

The raw data supporting the conclusions of this article will be made available by the authors, without undue reservation.
